# Clinical risk assessment in early pregnancy for preeclampsia in nulliparous women: A population based cohort study

**DOI:** 10.1371/journal.pone.0225716

**Published:** 2019-11-27

**Authors:** Anna Sandström, Jonathan M. Snowden, Jonas Höijer, Matteo Bottai, Anna-Karin Wikström

**Affiliations:** 1 Clinical Epidemiology Division, Department of Medicine Solna, Karolinska Institutet, Stockholm, Sweden; 2 Department of Women’s and Children’s Health, Uppsala University, Uppsala, Sweden; 3 School of Public Health, Oregon Health and Science University-Portland State University, Portland, Oregon, United States of America; 4 Unit of Biostatistics, Institute of Environmental Medicine, Karolinska Institutet, Stockholm, Sweden; Ospedale dei Bambini Vittore Buzzi, ITALY

## Abstract

**Objective:**

To evaluate the capacity of multivariable prediction of preeclampsia during pregnancy, based on detailed routinely collected early pregnancy data in nulliparous women.

**Design and setting:**

A population-based cohort study of 62 562 pregnancies of nulliparous women with deliveries 2008–13 in the Stockholm-Gotland Counties in Sweden.

**Methods:**

Maternal social, reproductive and medical history and medical examinations (including mean arterial pressure, proteinuria, hemoglobin and capillary glucose levels) routinely collected at the first visit in antenatal care, constitute the predictive variables. Predictive models for preeclampsia were created by three methods; logistic regression models using 1) pre-specified variables (similar to the Fetal Medicine Foundation model including maternal factors and mean arterial pressure), 2) backward selection starting from the full suite of variables, and 3) a Random forest model using the same candidate variables. The performance of the British National Institute for Health and Care Excellence (NICE) binary risk classification guidelines for preeclampsia was also evaluated. The outcome measures were diagnosis of preeclampsia with delivery <34, <37, and ≥37 weeks’ gestation.

**Results:**

A total of 2 773 (4.4%) nulliparous women subsequently developed preeclampsia. The pre-specified variables model was superior the other two models, regarding prediction of preeclampsia with delivery <34 and <37 weeks, both with areas under the curve of 0.68, and sensitivity of 30.6% (95% CI 24.5–37.2) and 29.2% (95% CI 25.2–33.4) at a 10% false positive rate, respectively. The performance of these customizable multivariable models at the chosen false positive rate, was significantly better than the binary NICE-guidelines for preeclampsia with delivery <37 and ≥37 weeks’ gestation.

**Conclusion:**

Multivariable models in early pregnancy had a modest performance, although providing advantages over the NICE-guidelines, in predicting preeclampsia in nulliparous women. Use of a machine learning algorithm (Random forest) did not result in superior prediction.

## Introduction

Recent evidence suggests that the risk of the generally more severe preterm preeclampsia (delivery <37 weeks) can be substantially reduced by prophylactic use of aspirin from early pregnancy to a defined high-risk population [1, 2]. Delay in the diagnosis of preeclampsia further contributes significantly to maternal morbidity and mortality [3–6]. Thus, accurate prediction of preeclampsia to enable preventive treatment and optimised surveillance is an urgent priority.

According to the National Institute for Health and Excellence’s (NICE) and other current national guidelines clinical early pregnancy decision rules for detection of women at high-risk of developing preeclampsia, are based on maternal and medical history risk factors [7–9]. These risk factors are evaluated individually without being incorporated into combined multivariable models, resulting in poor prediction, characterized by low sensitivity and specificity [10–12].

Clinical risk prediction models with combined predictor variables, also including medical examinations, have been developed in recent years and has led to improved detection rates [13, 14]. The Fetal Medicine Foundation (FMF) has created predictive models using a limited number of maternal factors with addition of mean arterial pressure (MAP) [15]. More complex FMF models include various combinations of biophysical, such as uterine artery Doppler, and biochemical markers, not routinely performed in antenatal care [10]. Since detection rates and cut-off values have shown to vary between populations, depending on differences in healthcare systems, incidence of disease and overfitting of the original model, the performance of these models have to be validated in other populations [16–19]. It has been emphasized that the cost-effectiveness of these more complex models has to be established before widespread use in clinical practice [14, 20]. Evidence further suggests that when using a vast number of clinical predictive variables and MAP in a model for low-risk nulliparous women, uterine artery Doppler does not improve the predictive capacity for preeclampsia [13]. A recent systematic review points out the need for development of predictive models with the optimal combination of simple maternal factors [14] and the predictors included in the FMF model do not comprise all known maternal risk factors for preeclampsia [12].

Nulliparous women have higher risk of preeclampsia and the predictive capacity of both clinical decision rules and multivariable models are better for parous than nulliparous women [11, 20, 21]. Few predictive models have been designed for nulliparous women [14] and this group would largely benefit from an improved screening. The performance of multivariable maternal factor models with MAP compared to the NICE guidelines risk classification in nulliparous women has to be further explored.

Swedish antenatal care is free of charge and almost all women attend [22]. Information on well-recognised, less established and unknown risk factors for preeclampsia, including almost all of the maternal characteristics variables incorporated in the FMF model and more, are collected at the first visit. Construction of predictive models using this comprehensive range of Swedish maternal health care data has not yet been performed In recent years, advanced prediction techniques including Machine learning methods [23] (e.g., Random forest) [24] have been implemented in medicine [25, 26]. These methods use data-driven approaches to select maximized predictive models using objective criteria rather than relying on expert opinion, and no assumptions of linearity or arbitrary cut-points are needed. These approaches also enable consideration of a large number of candidate predictors and complex interactions are possible to handle. To our knowledge, predictive models in early pregnancy for preeclampsia using a machine learning method have not been performed to date.

### Study objective

The objective was to create multivariable predictive models using three different methodological approaches (a logistic regression models with pre-specified variables similar to the Fetal Medicine Foundation model including maternal variables and MAP, a backward selection model starting from the full suite of variables, and a Random forest model) and the NICE-guidelines, to identify nulliparous women at increased risk of preeclampsia, using detailed routinely collected information from early pregnancy in a Swedish setting.

## Material and methods

### Setting

Data were derived from the Stockholm-Gotland Obstetric Cohort, a population-based database with information automatically retrieved from the computerized medical record system in the Stockholm-Gotland counties in Sweden. The database contains detailed, prospectively collected demographic, medical, obstetrical and neonatal data from all antenatal, delivery and postnatal care units in the region. Information is routinely entered into the medical records by midwifes or physicians in a standardized way. Approximately one fourth of all 115 000 annual births in Sweden occurs in the seven hospitals in the region.

### Study population

**Fig 1:**
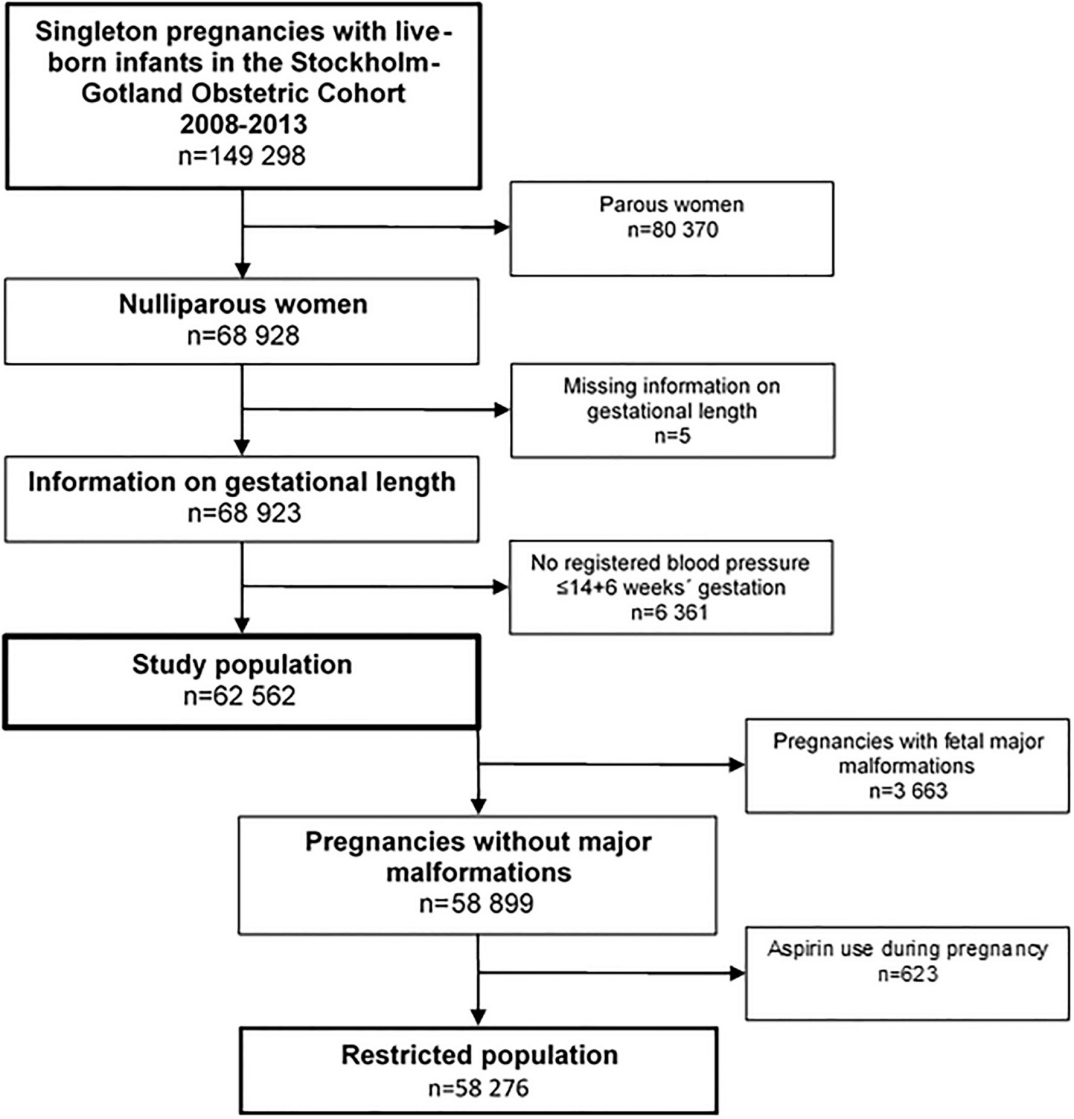
Flowchart of 149 298 included pregnancies of women who were delivering in the Stockholm-Gotland Counties of Sweden 2008–2013.

Live-born births between January 1^st^, 2008 and December 31^st^, 2013 were included in the cohort of 149 298 singleton pregnancies. The population was restricted to pregnancies of nulliparous women delivered from gestational week 22. Pregnancies of women without information on gestational length or without notation of blood pressure before 15 weeks’ gestation were excluded. The final study population included 62 562 pregnancies (Fig 1). We were also interested in predicting preeclampsia in pregnancies without major anomalies and among women not receiving aspirin, since this can alter the performance of the predictive models. For sensitivity analysis, pregnancies with major fetal malformations or maternal use of aspirin during pregnancy were excluded, giving a restricted population of 58 276 pregnancies (Fig 1).

### Data sources

The pregnancies in the Stockholm-Gotland Obstetric Cohort were individually linked using the person-unique national registration numbers with the National Patient Register [27] and the Swedish Prescribed Drug Register [28]. The National Patient Register includes International Classification of Diseases (ICD) diagnoses on inpatient admissions and outpatient visits. The Swedish Prescribed Drug Register holds data on all prescribed substances, ATC-code (Anatomical Therapeutic Chemical classification) and date of purchase for all dispensed drugs in the outpatient population.

### Study variables

#### Outcome

Diagnosis of preeclampsia was the key variable, classified according to the Swedish version of ICD-10 codes (O140, O141, O149 or O15) by the responsible doctor during pregnancy or at discharge, and was retrieved from the National Patient Register. Preeclampsia was defined as hypertension (blood pressure ≥140 mmHg and/or diastolic blood pressure ≥90 mmHg two times with an interval of at least 4 hours), combined with proteinuria (≥ 0.3 g/24 hours or 2+ on a dipstick testing) occurring after 20 weeks’ gestation. In order to fulfill our definition of preeclampsia, there had to be one diagnosis in the inpatient register or two in the outpatient register, where the date of the first diagnosis was used.

Accuracy of prediction of preeclampsia was quantified by detection rates (i.e. sensitivity) of diagnosis of preeclampsia; 1) *overall*: during pregnancy, 2) *early-onset*: with delivery <34 weeks, 3) *preterm*: with delivery <37 weeks and 4) *term*: with delivery ≥37 weeks.

#### Candidate predictors

At the first visit to antenatal care, around gestational week 10, the woman is interviewed about her social, reproductive and medical background, and medical examinations are performed. The routinely collected information from this visit were included in this study as 36 candidate predictors for preeclampsia in the predictive models, presented in Table 1.

**Table 1:** Predictive variables routinely collected at first visit to antenatal care in the study population of 62 562 nulliparous women.

Predictive variables	No preeclampsia n = 59 789 (95.6%)		Overall preeclampsia n = 2 773 (4.4%)		P-value*	Preterm (< 37 w) preeclampsia n = 497 (0.8%)		P-value**
**Gestational length first examination**, weeks ^a^	9.6	(1.9)	9.6	(1.9)	0.022	9.3	(1.9)	<0.001
**Maternal age**, years ^a^	29.3	(5.0)	29.9	(5.3)	<0.001	30.3	(5.8)	<0.001
**BMI,** kg/m^2^ ^a^	23.4	(4.0)	25.1	(4.9)	<0.001	24.7	(5.1)	<0.001
Missing (n)	2 066		92			11		
**Mean Arterial Pressure (MAP)**, mmHg ^a^	81.5	(8.0)	86.3	(9.1)	<0.001	86.7	(9.6)	<0.001
**Capillary glucose**, mmol/L ^a^	5.5	(1.1)	5.6	(1.1)	<0.001	5.7	(1.2)	0.002
Missing (n)	4 905		238			45		
**Protein in urine**, dipstick 0–3 ^a^	0.03	(0.2)	0.05	(0.3)	<0.001	0.06	(0.3)	0.001
Missing (n)	9 089		407			61		
**Hemoglobin (Hb)**, g/L ^a^	127.8	(10.3)	129.5	(10.5)	<0.001	130.0	(10.4)	<0.001
Missing (n)	4 462		221			42		
**Previous miscarriage** (n) ^a^	0.23	(0.57)	0.26	(0.60)	0.017	0.25	(0.62)	0.537
**Previous ectopic pregnancy** (n) ^a^	0.012	(0.12)	0.013	(0.13)	0.529	0.014	(0.13)	0.635
**Infertility duration**, years ^a^	0.40	(1.2)	0.54	(1.5)	<0.001	0.66	(1.8)	<0.001
**Family situation** n (%)					0.241			0.298
Single	1 225	(2.05)	72	(2.60)		16	(3.22)	
Living together with partner	54 996	(91.98)	2 533	(91.35)		455	(91.55)	
Other	3 203	(5.36)	149	(5.37)		23	(4.63)	
Missing (n)	365	(0.61)	19	(0.69)		3	(0.60)	
**Region of birth** n (%)					<0.001			0.164
Sweden	44 306	(74.10)	2 172	(78.33)		373	(75.05)	
Nordic countries (except of Sweden)	951	(1.59)	41	(1.48)		5	(1.01)	
Europe (except of Nordic countries)	4 496	(7.52)	154	(5.55)		31	(6.24)	
Africa	1 891	(3.16)	109	(3.93)		27	(5.43)	
North America	394	(0.66)	13	(0.47)		2	(0.40)	
South America	1 003	(1.68)	36	(1.30)		8	(1.61)	
Asia	5 912	(9.89)	187	(6.74)		43	(8.65)	
Oceania	54	(0.09)	3	(0.11)		0	(-)	
Missing (n)	782	(1.31)	58	(2.09)		8	(1.61)	
**Smoking 3 months before pregnancy** n (%)					0.867			0.054
<10	5 173	(8.65)	237	(8.55)		27	(5.43)	
≥10	4 333	(7.25)	190	(6.85)		32	(6.44)	
Missing (n)	361	(0.60)	16	(0.58)		4	(0.80)	
**Smoking at registration** n (%)					0.128			0.213
<10	2 071	(3.46)	76	(2.74)		14	(2.82)	
≥10	371	(0.62)	21	(0.76)		5	(1.01)	
Missing (n)	332	(0.56)	12	(0.43)		0	(-)	
**Snuff 3 months before pregnancy** n (%)	2 142	(3.58)	110	(3.60)	0.288	20	(4.02)	0.610
**Snuff at registration** n (%)	624	(1.04)	35	(1.26)	0.271	4	(0.80)	0.586
**Alcohol consumption 3 months before registration** n (%)					<0.001			0.004
≤ Once a week	20 226	(33.83)	929	(33.50)		149	(29.98)	
> Once a week	9 485	(15.86)	349	(12.52)		61	(12.27)	
Missing (n)	3 665	(6.13)	172	(6.20)		27	(5.43)	
**Alcohol consumption at registration** n (%)					0.634			0.160
≤ Once a week	445	(0.74)	19	(0.69)		8	(1.61)	
> Once a week	122	(0.20)	3	(0.11)		1	(0.20)	
Missing (n)	3 655	(6.11)	162	(5.84)		29	(5.84)	
**Family history of preeclampsia** n (%)	150	(0.25)	18	(0.65)	<0.001	5	(1.01)	0.001
**Family history of hypertension** n (%)	10 034	(16.78)	634	(22.86)	<0.001	116	(23.34)	<0.001
**Infertility** n (%)					0.006			0.870
Without treatment	3 997	(6.69)	201	(7.25)		36	(7.24)	
Ovary stimulation	885	(1.48)	48	(1.73)		9	(1.81)	
IVF	3 979	(6.66)	225	(8.11)		35	(7.04)	
**Cardiovascular disease** n (%)	780	(1.30)	49	(1.77)	0.037	8	(1.61)	0.578
**Endocrine disease** n (%)	2 983	(4.99)	174	(6.27)	0.002	34	(6.84)	0.066
**Pre-existing diabetes** n (%)	264	(0.44)	62	(2.24)	<0.001	21	(4.23)	<0.001
**Thrombosis** n (%)	417	(0.70)	16	(0.58)	0.454	2	(0.40)	0.434
**Psychiatric disease** n (%)	5 542	(9.27)	271	(9.77)	0.372	51	(10.26)	0.455
**SLE** n (%)	68	(0.11)	3	(0.11)	0.932	1	(0.20)	0.560
**Epilepsy** n (%)	367	(0.61)	18	(0.65)	0.816	3	(0.60)	0.973
**Chronic hypertension** n (%)	260	(0.43)	43	(1.55)	<0.001	12	(2.41)	<0.001
**Mb Crohn/Ulcerous colitis** n (%)	514	(0.86)	19	(0.69)	0.328	5	(1.01)	0.707
**Lung disease or asthma** n (%)	4 915	(8.22)	272	(9.81)	0.003	50	(10.06)	0.151
**Chronic kidney disease** n (%)	276	(0.46)	27	(0.97)	<0.001	10	(2.01)	<0.001
**Hepatitis** n (%)	486	(0.81)	22	(0.79)	0.911	7	(1.41)	0.137
**Gynaecological disease or operation** n (%)	11 329	(18.95)	528	(19.04)	0.903	102	(20.52)	0.370
**Recurrent urinary tract infections** n (%)	9 081	(15.19)	397	(14.32)	0.211	66	(13.28)	0.243
**Blood group** n (%)					0.124			0.032
0	21 039	(35.19)	930	(33.54)		147	(29.58)	
A	23 782	(39.78)	1 117	(40.28)		199	(40.04)	
AB	3 044	(5.09)	127	(4.58)		30	(6.04)	
B	7 344	(12.28)	369	(13.31)		71	(14.29)	
Missing (n)	4 580	(7.66)	230	(8.29)		50	(10.06)	

^a^ Mean at registration (standard deviation)

* Preeclampsia overall compared to no preeclampsia.

** Preterm preeclampsia compared to no preterm preeclampsia (no preeclampsia and term preeclampsia).

Gestational length was determined using the following hierarchy: a) date of embryo transfer, b) early first or early second trimester ultrasound, c) date of last menstrual period, and d) from postnatal assessment. Information on social factors (family situation and country of birth), smoking, snuff and alcohol habits as well as reproductive history (parity, previous miscarriage or ectopic pregnancy, assisted reproduction and infertility duration) are self-reported. Further, the women are interviewed about their medical history (including pre-existing chronic diseases). The definition of diabetes included pre-gestational diabetes type I and II. The collected information is registered in a standardized way either as tick boxes, pre-specified options, or as numbers. Family history of hypertension or preeclampsia is however registered as free text and two dichotomous variables (family history of hypertension and family history of preeclampsia) were constructed.

Maternal BMI (kg/m^2^) was calculated from self-reported height and measured or self-reported weight. Maternal blood pressure is measured by the midwife in supine position on the right upper arm using manual blood pressure equipment with a cuff size appropriate for arm circumference. Korotkoff V is used for diastolic blood pressure. The first recorded blood pressure <15 weeks was collected. Mean arterial pressure (MAP), defined as: (systolic blood pressure + (2 x diastolic blood pressure))/3, was calculated and used in the predictive models. Capillary blood sampling for plasma glucose and haemoglobin, venous sampling for blood group and urine dipstick test for protein is collected. All the candidate predictors were treated as continuous or categorized as presented in Table 1.

#### Restricted population

Occurrence of major malformation was defined as any recorded congenital anomaly in the National Patient Register (ICD-10 codes Q00–Q99), excluding minor malformations not reported to the Register of Birth Defects [29]. Use of aspirin during pregnancy was defined as purchased prescription of aspirin during pregnancy in the Swedish Prescribed Drug Register (a prescription is needed for aspirin for the doses indicated during pregnancy).

### Statistical methods

Statistical analyses were done with STATA 12 (StataCorp, College Station, TX, USA) for univariable and multivariable regression analyses. For Random Forest analyses, the statistical software package R (version 3.4.4, R Foundation for Statistical Computing, Vienna, Austria) was used. Chi-squared test and two sample t-test were used for comparing the variables in the study population in women who did and did not develop preeclampsia. In order to maximize the predictive power of our predictive models, we used three different multivariable statistical methods:

#### Pre-specified variables model

In this multivariable regression model for nulliparous women we used similar variables as in the FMF maternal factors and MAP model [11]. The included variables in the two models are specified in Table 2. For internal validation, we did a 10-fold cross-validation, using randomly allocated 90% of the data to generate a predictive model, and estimation of the risk of preeclampsia is then applied to the remaining 10% of the sample. This splitting procedure is repeated a large number of times and the performance of the model is then summarized.

**Table 2:** Variables included in the pre-specified model and the corresponding similar variables included in the Fetal Medicine Foundation’s (FMF) model with maternal characteristics and mean arterial pressure for nulliparous women.

Variables included in the pre-specified model	Corresponding variables included in the FMF^a^-model
Family history of preeclampsia	Mother of the woman had preeclampsia
Country of birth	Racial origin
Method of conception	Method of conception
Gestational length at registration (Based on 1. date of embryo transfer, 2. early first or early second trimester ultrasound, 3. date of last menstrual period, and 4. from postnatal assessment)	Gestational length at registration (all based on first trimester ultrasound)
Maternal age	Maternal age
Height	Height
Weight	Weight
Smoking habits in early pregnancy	Smoking habits in early pregnancy
Pre-existing diabetes (Type I and II)	Diabetes type I
	Diabetes type II
Chronic hypertension	Chronic hypertension
Systemic lupus erythematosus	Systemic lupus erythematosus
	Anti-phospholipid syndrome
Mean arterial pressure (MAP)	Mean arterial pressure (MAP)

^a^ Fetal Medicine Foundation

#### Backward selection model

To select the best variables for this model for each outcome, we used backward selection on a multivariable logistic regression with an exclusion criterion of p-value more than 0.2. We submitted the 36 candidate predictors described above to this model-selection procedure. For internal validation, a 10-fold cross-validation was used.

#### Random forest model

We used Random forest [24], a machine learning method [23], which is an ensemble method making use of multiple decision trees. We submitted the same 36 candidate predictors employed in the backward selection procedure in the Random Forest approach. For each tree, a bootstrap sample was drawn, from which the tree was built. In order to get an unbiased estimate of the area under the receiver operating characteristic curve (AUC), the Out-of-Bag samples were used when predicting the probabilities of the outcomes.

#### NICE-guidelines

In addition to the multivariable models described above, we created a risk classification system based on the NICE-guidelines binary (high-risk: yes or no) clinical decision rule. Having a high-risk for preeclampsia according to the NICE-guidelines for nulliparous women in early pregnancy include any of the following risk factors: Chronic kidney disease, systemic lupus erythematosus, antiphospholipid syndrome (not included in our NICE-guidelines model), type 1 or type 2 diabetes, chronic hypertension, age 40 or older, BMI 35 or more at registration, and family history of preeclampsia [9].

#### Missing values

To increase the power and minimise selection bias we used single-chained imputation with mean values for missing observations for the variables with missing information (Table 1).

#### AUC

The AUC for the three multivariable methods were calculated using bias corrected bootstrap confidence intervals. The detection rate of preeclampsia at a 10% fixed false positive rate (FPR) was calculated with a 95% confidence interval (CI) using the Clopper-Pearson method.

## Results

In the study population of 62 562 nulliparous women, 2 773 (4.4%) developed preeclampsia during pregnancy. In total 216 (0.3%) developed preeclampsia with delivery <34 weeks, 497 (0.8%) developed preeclampsia with delivery <37 weeks and 2 276 (3.6%) developed preeclampsia with delivery ≥37 weeks, respectively. Aspirin was used by 623 (1.1%) of the women with non-anomalous pregnancies (Fig 1).

The social, reproductive and medical background and medical examination variables from the first visit in antenatal care are presented in Table 1, stratified into women who did not develop preeclampsia, women who developed preeclampsia overall and women who developed preeclampsia with delivery <37 weeks. Women who developed preeclampsia overall were significantly (p-value of <0.05) older, had higher BMI, longer infertility duration, more often having assisted reproduction and previous miscarriages compared to women who did not develop preeclampsia. A family history of hypertension or preeclampsia, being born in Africa or in Sweden and chronic diseases were more common among women in the preeclampsia group. Medical examinations at first visit displayed increased capillary glucose levels, rates of proteinuria, haemoglobin levels and MAP among women who developed preeclampsia.

The variable used in the backward selection analyses for prediction of preeclampsia with delivery at <34, <37 and ≥37 weeks with 10-fold cross validation are listed in S1 Table.

**Fig 2:**
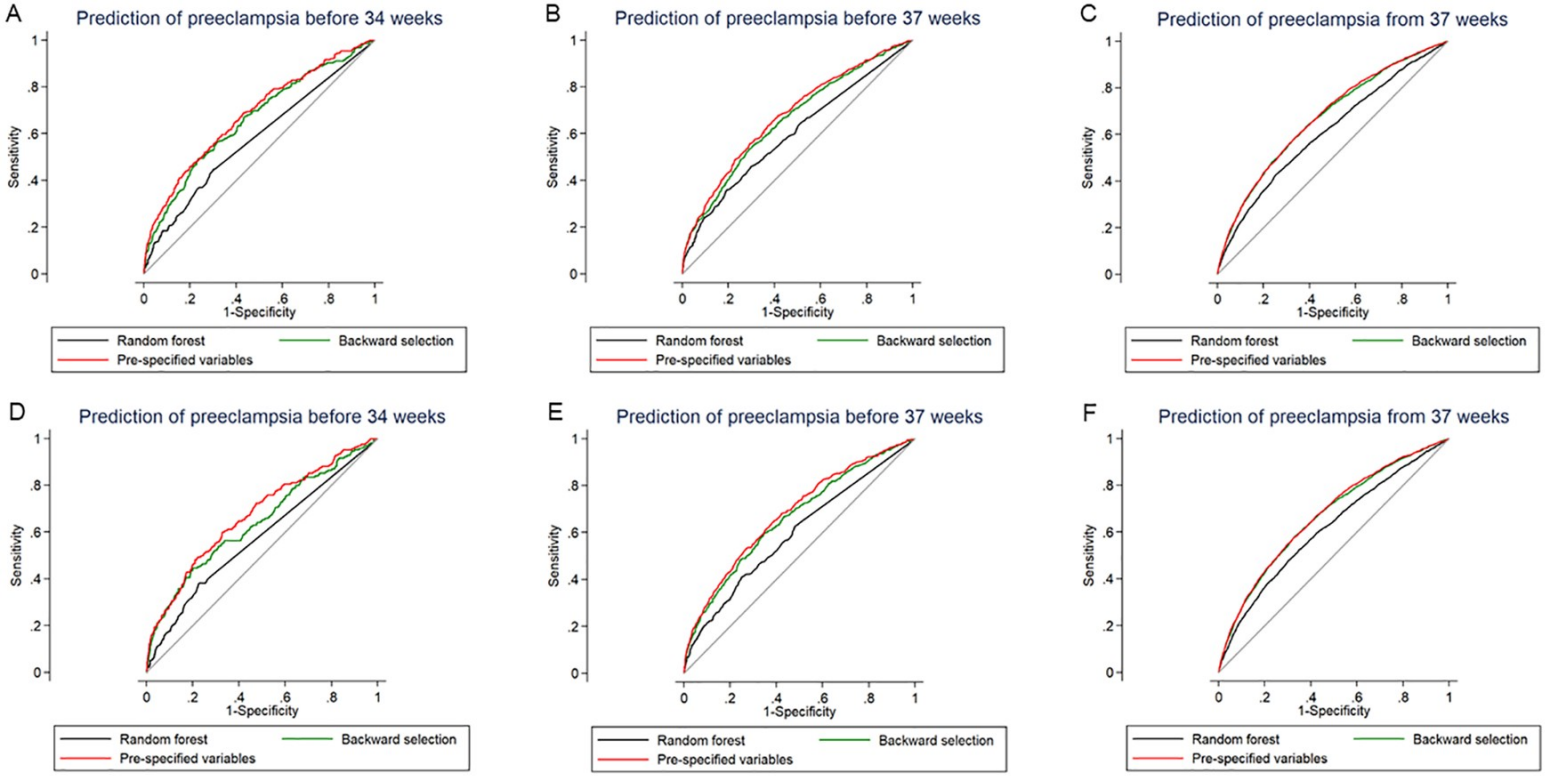
Prediction of preeclampsia in the total study population (A-C) and in the restricted population of pregnancies without major malformations or treatment with aspirin (D-F) before 34 (A, D), before 37 (B, E) and from 37 (C, F) weeks’ gestation based on pre-specified variables, backward selection and Random forest methods.

The receiver operating characteristic (ROC)-curves of the variables ability to predict preeclampsia at <34, <37 and ≥37 weeks with the three different multivariable methods are given in Fig 2. Fig 2A–2C include the total study population and Fig 2D–2F present the restricted population without pregnancies with major malformations or treatment with aspirin.

The AUC to predict preeclampsia in the total and the restricted populations for the three outcomes are given in Table 3. Based on the variables included, regardless of any of the three multivariable methods used for prediction of preeclampsia at <34, <37 or ≥37 weeks, the AUC did not reach more than 0.68, indicating uniformly low-to-moderate predictive ability (Table 3).

**Table 3:** Area under the ROC curve and sensitivity for prediction of preeclampsia with delivery at <34, <37 and ≥37 weeks’ gestation based on pre-specified variables, backward selection and Random forest methods.

	Total study population n = 62 562				Restricted study population^c^ n = 58 276			
Predictive method	Area under the ROC^a^ curve	(95% CI)	Sensitivity for 10% FPR^b^	(95% CI)	Area under the ROC^a^ curve	(95% CI)	Sensitivity for 10% FPRb	(95% CI)
**Prediction of preeclampsia <34 weeks**								
Pre-specified variables	0.68	(0.64–0.72)	30.6	(24.5–37.2)	0.67	(0.63-0-72)	28.8	(22.1–36.3)
Backward selection	0.66	(0.62–0.70)	26.9	(21.1–33.3)	0.64	(0.60–0.69)	28.8	(22.1–36.3)
Random forest	0.58	(0.54–0.62)	18.5	(13.6–24.4)	0.57	(0.53–0.62)	17.6	(12.2–24.2)
**Prediction of preeclampsia <37 weeks**								
Pre-specified variables	0.68	(0.65–0.70)	29.2	(25.2–33.4)	0.68	(0.65-0-71)	29.3	(25.0–34.0)
Backward selection	0.66	(0.63–0.68)	25.8	(22.0–29.8)	0.66	(0.63–0.69)	27.9	(23.6–32.5)
Random forest	0.60	(0.57–0.63)	24.3	(20.6–28.4)	0.60	(0.57–0.62)	21.4	(17.5–25.7)
**Prediction of preeclampsia ≥37 weeks**								
Pre-specified variables	0.67	(0.66–0.69)	28.1	(26.3–30.0)	0.67	(0.66–0.68)	27.6	(25.7–29.5)
Backward selection	0.67	(0.66–0.68)	28.2	(26.4–30.1)	0.67	(0.66–0.68)	27.5	(25.6–29.5)
Random forest	0.61	(0.60–0.62)	22.4	(20.7–24.2)	0.62	(0.60–0.63)	22.7	(20.9–24.5)

^a^ ROC: Receiver operating characteristic

^b^ FPR: False-positive rate

^c^ Restricted study population: Pregnancies without major malformations or treatment with aspirin

The sensitivity at a FPR of 10% for preeclampsia <34 and <37 weeks were superior in the groups of pre-specified variables. For detection of preeclampsia with delivery ≥37 weeks, the best performing models were the pre-specified variables and the backward selection, compared to the Random forest model (Table 3).

When using the binary NICE-guidelines risk classification system for identifying women at risk of preeclampsia in our population, 5.8% of all nulliparous women would be classified as high risk (screen positive). The detection rate for preeclampsia with delivery <34 weeks would be 22.2% (95% CI 16.8–28.4), preeclampsia with delivery <37 weeks 19.5% (95% CI 16.1–23.3) and preeclampsia with delivery ≥37 weeks 12.2% (95% CI 10.9–13.7), all with a fixed FPR of about 5.5%. In our best performing models with a chosen FPR of 10%, the detection rate is higher for preterm and term preeclampsia, but with an overlapping CI for early onset preeclampsia, compared to the NICE-guidelines.

## Discussion

### Main findings

In this population-based cohort study of 62 562 nulliparous women, we found that using routinely collected information on well-known and less established or unknown risk factors from first visit to antenatal care as predictive variables generated a modest predictive capacity for preeclampsia, irrespective of type of multivariable statistical method used.

The prediction of preeclampsia with delivery <34, <37 or ≥37 weeks with the three different methods was similar with AUC’s of 0.58–0.68. The sensitivities at a fixed 10% FPR varied between 18.5–30.6%. The performance of the customizable multivariable risk prediction approach at the FPR of 10% was however significantly better than using the binary NICE-guidelines for preeclampsia with delivery <37 weeks’ and ≥37 weeks.

### Strengths and limitations

Given the nature of predictive research, this comprehensive set of fine-grained prospectively routinely collected variables, with generally a minimal level of missing values, collected on a large population represents a distinct strength, increasing the likelihood of accurate prediction [30]. The use of a database with automatically retrieved information from the computerized medical record system with standardized data registration, reduced erroneous data entry and transcription errors. There is no consensus regarding the best method for selection of variables for a predictive model [31]. The large number of preeclampsia cases enabled use of the full suite of the 36 variables in the predictive models giving more than ten events per candidate predictor for preeclampsia with delivery <37 and ≥37 weeks, and six events for preeclampsia with delivery <34 weeks. Too few events per candidate predictor has been described as a limitation in risk models for preeclampsia [32].

We internally validated the performance of the three multivariable predictive methods with 10-fold cross validation for the two logistic regression analyses and bootstrap in the Random forest analyses, preventing over-fitting observed in single-tree approaches. No external validation was carried out in the scope of our study, however, our inclusion criteria were precisely defined, facilitating future external validations.

Despite the richness of our data resources, data quality issues are nonetheless a concern. Potential misclassification of the mainly self-reported variables in the interview with the midwife could have occurred, but since the predictive models were based on routinely collected information, this would probably reflect the outcome of the models in the clinical setting. Since this is a retrospective study we could not influence the procedure of blood pressure examinations, where a potential misclassification bias by measurement errors could have been introduced. However, midwives have guidelines for conducting blood pressure examinations and a differential misclassification seems implausible.

Using the ICD-10 diagnosis instead of data from medical records to determine the diagnosis of preeclampsia could have introduced misclassification bias of the outcome. In order to improve the accuracy of the diagnosis, one diagnosis in the inpatient or two diagnoses in the outpatient register was required. The Swedish version of ICD-10 diagnoses, still define preeclampsia with mandatory proteinuria, which is less sensitive but more specific compared to current international recommendations of the diagnosis [11, 20]. With few exceptions, previous predictive models have used or done sensitivity analyses with the same definition [13, 14]. Overall rates of preeclampsia in nulliparous women in our study were consistent with previous populations from western countries [13, 33].

The effect of aspirin treatment during pregnancy has not been taken into account in the creation of previous predictive models for preeclampsia [11, 14]. When adjusting for the assumed effect of aspirin (i.e. the reduced risk of preterm preeclampsia in high risk women) in one study, the detection rates for preeclampsia were slightly, but not significantly reduced [10]. To address this in our study, we made a sensitivity analysis in a restricted cohort excluding pregnancies with use of aspirin and also major malformations, without significant alterations of the model’s predictive capacity. This could possibly reflect the poor selective performance of women for use of aspirin in current Swedish clinical practice.

### Interpretation

In univariate analyses we found that well established risk factors for preeclampsia, such as increasing BMI and maternal age, assisted reproduction, country of birth, chronic hypertension, pre-exciting diabetes, chronic kidney disease and family history of preeclampsia were individually associated with development of preeclampsia [12, 20, 34, 35]. Compared to women without preeclampsia, women who developed preeclampsia had higher MAP and haemoglobin level in early pregnancy in accordance with previous knowledge [13, 36–39].

Prior studies, using the FMF-model with maternal factors and MAP for mixed parities at a 10% FPR have demonstrated a sensitivity of 47–59% and 37–43% for preterm and term preeclampsia, respectively [10, 15, 21, 40]. Nulliparous women have higher risk of preeclampsia, and no marker of individual risk based on previous obstetric history [20, 41]. The predictive capacity of both clinical guidelines and multivariable models are better for parous than nulliparous women, and there are only a few predictive studies on nulliparous women separately, making the evidence gap more pressing for this group [11]. Previous predictive studies on nulliparous women, using different inclusion criteria and combinations of maternal factors, demonstrates an AUC of 0.71 and sensitivity of 31–37% with a FPR of 10–11.5% for preeclampsia overall [11, 13]. For preeclampsia with delivery <37 weeks, an AUC of 0.76 and sensitivity of 34–36% at a FPR of 5–11.5% has been reported, indicating better prediction than in our study [11, 42].

Defining predictive performance according to how preeclampsia prediction is used in practice by a clinician, we argue that using a customizable multivariable risk-prediction approach is superior to the binary NICE classification system, which here results in a fixed false-positive rate of 5.5%. One advantage of using a multivariable risk-prediction framework to address this question is that it more flexible to investigator/clinician choices about optimal sensitivity/specificity. The NICE-guidelines’ predictive capacity for preeclampsia in our cohort of nulliparous women was inferior compared to our multivariable models tested at a FPR of 10%, in accordance with previous knowledge, but also inferior compared to the NICE-guidelines performance in other populations [11, 13]

The generally lower sensitivities in our study compared to other nulliparous populations could be due to several factors: differences in demographic factors, the population-based routinely collected material in our study compared to a prospective design with volunteer enrolment, and different methodological approaches. Further, internal validation with bootstrapping and 10-fold cross validation used in our study generally tend to reduce the risk of overestimating the predictive capacity of a predictive model [19, 43]. The lower detection rate could also reflect a need for validation of maternal health care data and possibly improved collection of data.

First trimester screening studies for the risk of preeclampsia in different clinical settings is emphasised by the International Society for the Study of Hypertension in pregnancy (ISSHP)[20] and to our knowledge, no previous multivariable predictive study has been evolved or evaluated in a Swedish setting.

Machine learning methods [23] as Random forest [24] have been demonstrated to yield accurate prediction in some biomedical applications [25], but in this study resulted in prediction that was less accurate than the logistic regression models. There are many potential explanations for this finding e.g., overreliance on data-based considerations, over-fitting, too many features unrelated to the outcome that swamp the true signals [44].

## Conclusion

The capacity of our multivariable predictive models for preeclampsia with delivery <34, <37 and ≥37 weeks in nulliparous women was overall modest, but it is worth noting that these models have advantages as compared to the binary NICE-guidelines risk classification. The logistic regression models performed better than models using Random forest, indicating that future work on this topic should continue to incorporate clinical expertise as well as newer prediction approaches.

The models to predict preeclampsia reported here provide a step towards a personalised risk score for preeclampsia in nulliparous women, a group that would largely benefit from an improved screening and for whom only a few predictive models have been designed [14].

To improve overall accuracy and detection of cases, the variables for the clinical model has to be validated and potentially with the addition of biochemical or biophysical markers.

## Supporting information

S1 Table Variables used in the backward selection models for prediction of preeclampsia with delivery at <34, <37 and ≥37 weeks’ gestation with 10-fold cross validation. (DOCX)
