# Supplementary material for: Clinical risk assessment in early pregnancy for preeclampsia in nulliparous women: A population based cohort study
Source: PLoS One. 2019 Nov 27;14(11):e0225716. doi: 10.1371/journal.pone.0225716 (PMC6881002; doi:10.1371/journal.pone.0225716)
Supplement: S1 Table — (DOCX) [file pone.0225716.s001.docx]

**S1 Table.** Variables used in the backward selection models for prediction of preeclampsia
with delivery at <34, <37 and ≥37 weeks’ gestation with 10-fold cross validation.

|  | **Prediction of preeclampsia**  **<34 weeks** | **Prediction of preeclampsia**  **<37 weeks** | **Prediction of preeclampsia**  **≥37 weeks** |
| --- | --- | --- | --- |
| Maternal age | X | X | X |
| BMI | X | X | X |
| Mean Arterial Pressure (MAP) | X | X | X |
| Protein in urine | X | X | X |
| Infertility treatment | X | X | X |
| Diabetes | X | X | X |
| Blood group | X | X | X |
| Alcohol consumption at registration | X | X | X |
| Gestational length at registration | X | X |  |
| Capillary glucose |  | X | X |
| Hemoglobin |  | X | X |
| Infertility duration | X | X |  |
| Family history of preeclampsia |  | X | X |
| Family history of hypertension |  | X | X |
| Alcohol consumption 3 months before registration |  | X | X |
| Chronic kidney disease | X | X |  |
| Family situation | X |  |  |
| Smoking 3 months before pregnancy | X |  |  |
| Smoking at registration |  |  | X |
| Snuff 3 months before pregnancy | X |  |  |
| Snuff at registration |  |  | X |
| Region of birth |  |  | X |
| Hepatitis | X |  |  |
| Morbus Chron/Ulcerous colitis |  |  | X |
| Psychiatric disease | X |  |  |
